# Flow Cytometric Features of B- and T-Lmphocytes in Reactive Lymph Nodes Compared to Their Neoplastic Counterparts in Dogs

**DOI:** 10.3390/vetsci10060374

**Published:** 2023-05-26

**Authors:** Fulvio Riondato, Alessia Poggi, Barbara Miniscalco, Federica Sini, Laura Marconato, Valeria Martini

**Affiliations:** 1 Department of Veterinary Sciences, University of Turin, Largo Braccini 2, Grugliasco, 10095 Turin, Italy; alessia.poggi@unito.it (A.P.); barbara.miniscalco@unito.it (B.M.); federica.sini@unito.it (F.S.); 2 Department of Veterinary Medical Sciences, University of Bologna, Via Tolara di Sopra, 43, Ozzano dell’Emilia, 40064 Bologna, Italy; laura.marconato@unibo.it; 3 Department of Veterinary Medicine and Animal Sciences, University of Milan, Via Dell’Università 6, 26900 Lodi, Italy; valeria.martini@unimi.it

**Keywords:** dog, flow cytometry, lymph nodes, CD5, CD21, Ki-67

## Abstract

**Simple Summary:**

In-depth knowledge of non-neoplastic patterns is fundamental when diagnosing neoplasia. In the present study, we described the flow cytometric (FC) features of B- and T-lymphocytes in 42 canine reactive lymph nodes and 36 lymphomas. Proliferative activity (Ki67%) in reactive lymph nodes was also reported. Reactive lymph nodes were composed of a mixed population of small and large T (CD5+) and B (CD21+) cells. Small T-cells were larger in size than small B-cells and large T-cells were larger than large B-cells. Large B-cells were <20% in reactive lymph nodes and >20% in B-cell lymphomas and showed a larger size in lymphomas than in reactive lymph nodes. Large T-cells were ≤4% in reactive lymph nodes and >4% in T-cell lymphomas and showed higher CD5 expression in lymphomas compared to reactive lymph nodes. We report, for the first time, a subset of small CD5+CD21+dim cells in reactive lymph nodes, whose biological role has yet to be defined. Ki-67% values were higher than those reported in normal lymph nodes, and largely overlapped with those reported in lymphomas.

**Abstract:**

An in-depth knowledge of non-neoplastic patterns is fundamental to diagnose neoplasia. In the present study, we described the flow cytometric (FC) cell size (FSC) and fluorescence intensity (MFI) of B- and T-lymphocytes in 42 canine reactive lymph nodes and 36 lymphomas. Proliferative activity (Ki67%) in reactive lymph nodes was also reported. Reactive lymph nodes were composed of a mixed population of small and large T (CD5+) and B (CD21+) cells. Small T-cells were larger in size than small B-cells, and large T-cells were larger than large B-cells. Small T-cells were composed of CD5+CD21− and CD5+CD21+dim subpopulations. Large B-cells were <20% in reactive lymph nodes and >20% in lymphomas and showed a higher FSC in lymphomas than in reactive lymph nodes. Large T-cells were <4% in reactive lymph nodes and >4% in lymphomas and showed a higher CD5 MFI in lymphomas (if expressed) compared to reactive lymph nodes. A subset of CD5+CD21+dim lymphocytes was recognized in addition to CD5+CD21- and CD5−CD21+ cells. In T-zone lymphomas, neoplastic cells had higher FSC and CD21 MFI values than small CD5+CD21+dim cells in reactive lymph nodes. Ki67% values were higher than those reported in normal lymph nodes, and largely overlapped with those reported in low-grade lymphomas and partially in high-grade lymphomas. Our results may contribute to making a less operator-dependent FC differential between lymphoma and reactive lymph nodes.

## 1. Introduction

In recent decades, flow cytometry (FC) has become popular in veterinary medicine, and it is currently widely applied to diagnose and characterize canine lymphomas. Nevertheless, the analysis of FC data remains strongly operator-dependent [1,2], and the criteria to diagnose lymphoma vary among published studies [3,4,5,6].

An in-depth knowledge of the composition of normal or non-neoplastic tissues is mandatory to identify neoplastic conditions and obtain an accurate diagnosis of lymphoma. In dogs, the percentage of lymphoid subsets and Ki67 positive cells (Ki67%) in normal lymph nodes (LNs), assessed via FC, has been reported [7,8]. Despite their relevance, these data are not sufficient to discriminate between lymphomatous and non-neoplastic LNs, since both the cell size and level of antigen expression (as assessed by median fluorescence intensity (MFI) of antibody-labelled cells) should be taken into account [9].

The aim of the present study was to provide data on FC cell size and the MFI of CD21+ and CD5+ cells in reactive LNs in dogs, and to describe possible differences in their neoplastic counterpart. In addition, the proliferative activity (Ki67%) of reactive LNs was investigated.

## 2. Materials and Methods

The FC database of the diagnostic service of the Veterinary Teaching Hospital, University of Turin, was retrospectively investigated from January 2018 to December 2021, and samples fulfilling the following mandatory inclusion criteria were extracted: (1) FC analysis of an aspirate obtained from an enlarged peripheral LN; (2) sample acquired with a BD Accuri C6 (Beckton Dickinson, San Josè, CA, USA) flow cytometer; (3) concomitant assessment of CD5 and CD21 expression in a single tube, with anti-CD5 FITC-conjugated antibody (clone YKIX322.3, Biorad, Oxford, UK) and anti-CD21 AlexaFluor647-conjugated antibody (clone CA2.1D6, Biorad); (4) the availability of a cytological smear and raw FC data for review.

Among the extracted cases, samples were classified as “reactive LNs” and eventually included in the study if a reactive pattern was found at cytological evaluation (prevalence of small-sized mature lymphocytes, and less represented medium- and large-sized lymphoid cells, macrophages and plasma cells) [10,11] and at least two of the following criteria were fulfilled: (1) the final identification of any systemic or local cause of nodal reactive hyperplasia; (2) histopathologic diagnosis of nodal reactive hyperplasia; (3) polyclonal pattern of both TCR and IgG genes confirmed via PCR for Antigen Receptor Rearrangement (PARR); (4) no clinical or laboratory finding indicative of lymphoma for 6 months after FC analysis.

In addition, samples processed in the same period with the same protocol but finally diagnosed as lymphoma via histopathology, and classified according to the WHO system, were included.

**Figure 1:**
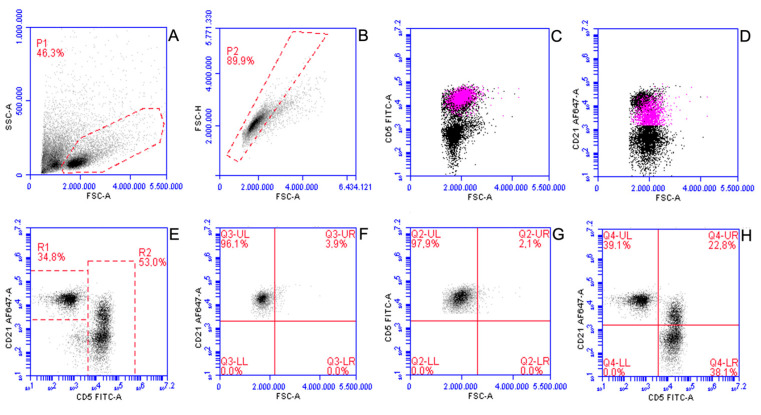
Gating strategy applied to analyze flow cytometric features of B- and T-lymphocytes within reactive lymph nodes in 42 dogs. (**A**) All events are displayed; a gate (P1) was set on FSC versus SSC scattergram to exclude platelets and debris. (**B**) P1 events are displayed; a second gate (P2) was set on FSC-H versus FSC-A scattergram to exclude doublets. (**C**,**D**) P2 events are displayed; CD5+CD21+ cells (pink dots) are undistinguishable from remnant CD5+ small cells (**C**), whereas they are clearly discriminated from remnant CD21+ cells. (**E**) P2 events are displayed; two gates were set to analyze CD21+CD5- cells (R1) and CD5+ cells (R2). (**F**,**G**) R1 (**F**) and R2 (**G**) events are displayed, respectively; cells were discriminated into small (Q3-UL and Q2-UL) and large (Q3-UR and Q2-UR). The discriminator for cell size was arbitrarily fixed based on FSC of circulating CD21+ and CD5+ lymphocytes in healthy dogs by including all positive events in Q3-UL and Q2-UL, respectively. (**H**) Q2-UL and Q3-UL cells were combined in a final scattergram; three subsets were identified: small CD5-CD21+ (Q4-UL), small CD5+CD21- (Q4-LR) and small CD5+CD21+ (Q4-UR); discriminators were set based on fluorescence of unstained cells.

For the specific purposes of the present study, all raw FC data were re-analysed by an experienced flow-cytometrist (FR) with dedicated software (CFlow Plus, Beckton Dickinson). All LN aspirates were sampled and suspended in RPMI-containing tubes, delivered to the laboratory and processed for FC within 24 h of sampling according to previousy published protocols [9]. In brief, up to 5 × 10^5^ cells from the cell suspension were incubated (20 min at 4 °C) with both antibodies and a blocking solution containing fetal bovina serum (FBS). An ammonium-based lysis buffer was added to remove contaminant erythrocytes and after 5 min of incubation at 4 °C, the tubes were centrifuged and supernatant discarded. Cell pellet was then washed and resuspended in PBS and acquired. A second tube was processed with the same protocol, without the inclusion of antibodies, to serve as a negative control. Propidium iodide was used in a separate tube to check viability of gated cells. Staining for Ki67 was performed in a subset of cases, according to previous published protocols [12]. In brief, cells were permeabilized using a commercial kit (Leucoperm, Bio-Rad, Hercules, CA, USA) according to manufacturer’s instructions, modified by the addition of an intermediate incubation (10 min at 4 °C) with 500ul of frozen methanol. After a washing step, the cell pellet was resuspended in the permeabilizing reagent and divided into two tubes. An anti-Ki67 antibody (clone MIB-1, Dako, Glostrup, Denmark) or isotypic antibody, both FITC-conjugated, were respectively added to the two tubes. After incubation for 30 min at 4 °C, cells were washed and resuspended with PBS and acquired. Ki67% was calculated on all intact cells (Figure S1). All samples were acquired with the same instrument and the same compensation matrix was applied to all analyses. A minimum of 10,000 intact cells per tube were acquired. The gating strategy for reactive LNs is shown in Figure 1.

The following data were recorded for each cellular population identified: percentage, median cell size (FSC-A), and median fluorescence intensity of positive cells for each antibody used (CD5-MFI and CD21-MFI, respectively). FSC-A and MFI values of peripheral blood neutrophils in the two fluorescence channels (FL-1 for CD5 and FL-4 for CD21) were also recorded for each dog, and they were used for normalization of FSC-A and MFI data. A paired peripheral blood sample was available for each included case; normalization was calculated by dividing the median FSC or MFI of lymphocytes by the median FSC or MFI of the paired blood neutrophils. Neutrophils were used instead of lymphocytes because CD5 /CD21 labelling was not always available and to allow for a comparison of FSC data with those reported in previously published papers [13,14,15,16,17]. Normalized data (nFSC, CD5-nMFI and CD21-nMFI) were finally used for statistical analyses. When available, Ki67% was also recorded.

For lymphoma samples, nFSC, CD5-nMFI and/or CD21-nMFI were calculated after gating on the neoplastic cellular population.

All data were recorded in an electronic datasheet and descriptive statistics were calculated.

The normal distribution of data was investigated via Shapiro–Wilk test. Then, a Friedman’s test for k-related samples was applied to detect possible differences in nFSC and nMFI values among the different cellular populations within reactive LNs. Post-hoc analyses were performed with a Wilcoxon signed-rank test with Bonferroni correction for multiple comparisons. All analyses were performed with SPSS software for Windows, v28.0. Significance was set at *p* ≤ 0.05 for all tests.

Statistical analyses were not performed on lymphoma samples due to the low number of samples within each WHO category. Thus, only a gross inspection of raw data was performed.

## 3. Results

### 3.1. Reactive Lymph Nodes

Overall, 42 samples fulfilled our inclusion criteria for reactive LNs. All cases except two met the inclusion criterion n.4 and they were combined with n.1 in most of the cases and/or n. 2 or 3 (four cases). The remaining two cases were included because they met criteria n. 2 and 3. No cases met only criteria n.1 and 2. Additionally, no indication of corticosteroid administration in the month prior to sample collection was reported for any case.

Three populations of B cells and T cells were recognized: CD5+CD21-, CD5-CD21+, and CD5+CD21+ (Figure 1H; Q4-LR, Q4-UL and Q4-UR respectively). The latter was made of small sized cells that clustered with CD5+CD21- but not with CD5-CD21+ cells based on fluorescence intensity (Figure 1C,D), and were thus considered as a whole CD5+ population when reporting the percentage of small and large cells. CD45 labelling was available for all included cases and no CD45-negative events were identified. Additionally, multicolor tubes showing CD3 and CD45 expression on CD5+CD21+ cells were carried out in one case (Figure S2). 

Descriptive statistics of percentage, nFSC, CD21-nMFI and CD5-nMFI for the different cellular populations are listed in Table 1.

**Figure 2:**
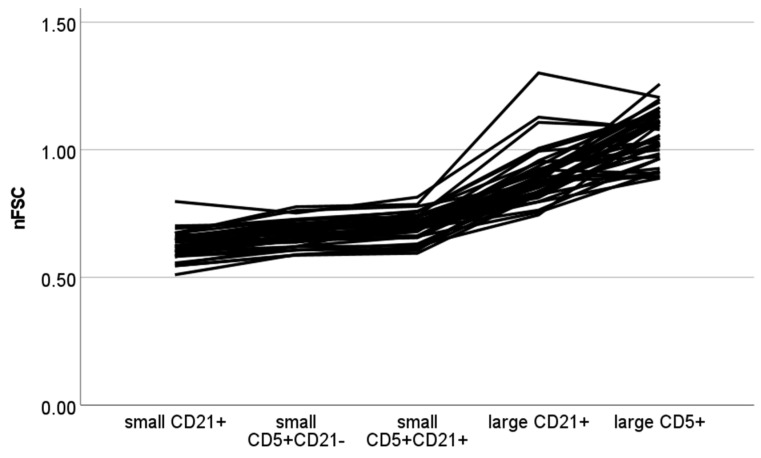
Normalized forward scatter (nFSC) values of different cellular populations detected via flow cytometry in lymph node aspirates from 42 dogs with nodal reactive hyperplasia. Each color represents a single case. Normalization was obtained by dividing median FSC of lymphocyte subset by median FSC of neutrophils in paired blood samples.

The results of the nFSC are shown in Figure 2. A statistically significant difference between populations was found (*p* < 0.001). Small CD21+ cells had a significantly lower nFSC than small CD5+CD21+, large CD21+ and large CD5+ cells (*p* < 0.001 for all paired contrasts). Small CD5+CD21- cells had a significantly lower nFSC than large CD21+ and CD5+ cells (*p* < 0.001 for both paired contrasts). Small CD5+CD21+ cells had a significantly lower nFSC than large CD21+ and CD5+ cells (*p* = 0.009 and *p* < 0.001, respectively). The difference in nFSC between small CD5+CD21- and small CD5+CD21+ cells and between small CD5+CD21- and small CD21+ cells did not reach statistical significance (*p* = 0.058 for both paired contrasts).

**Figure 3:**
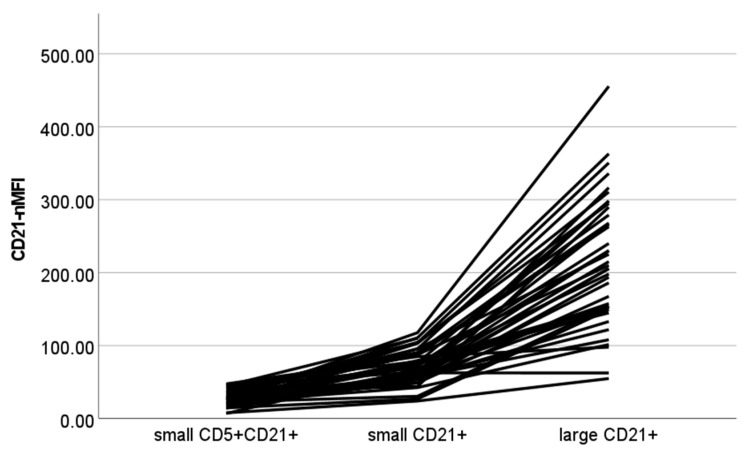
Normalized median fluorescence intensity of CD21+ cells (CD21-nMFI) values of different cellular populations detected via flow cytometry in lymph node aspirates from 42 dogs with nodal reactive hyperplasia. Each color represents a single case. Normalization was obtained by dividing MFI of lymphocyte subset by MFI of neutrophils in paired blood samples.

The results of CD21-nMFI are shown in Figure 3. A statistically significant difference among populations was found (*p* < 0.001), with all post-hoc contrasts showing significant results (*p* < 0.001 for all paired contrasts).

**Figure 4:**
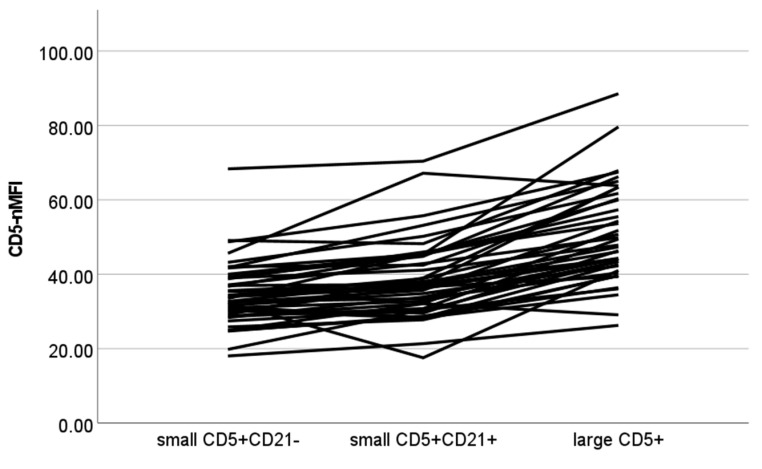
Normalized median fluorescence intensity of CD5+ cells (CD5-nMFI) values of different cellular populations detected via flow cytometry in lymph node aspirates from 42 dogs with nodal reactive hyperplasia. Each color represents a single case. Normalization was obtained by dividing MFI of lymphocyte subset by MFI of neutrophils in paired blood samples.

The results of CD5-nMFI are shown in Figure 4. A statistically significant difference among populations was found (*p* < 0.001), with all post-hoc contrasts showing significant results (*p* < 0.001 for all paired contrasts).

Ki67 expression was tested on 22 (52.4%) samples. Mean Ki67%, calculated for the whole population, was 4.6 ± 3.3% (median, 3.7%; min-max, 0.5–15.3%).

### 3.2. Lymphomas

Overall, 36 lymphoma samples fulfilled our inclusion criteria, including 31 B-cell lymphomas (20 diffuse large B-cell lymphoma (DLBCL), 6 marginal-zone lymphomas (MZL), 3 follicular lymphomas (FL), 1 B-cell lymphoblastic lymphoma and 1 Burkitt-like lymphoma) and 5 T-cell lymphomas (3 peripheral T-cell lymphomas (PTCL) and 2 T-zone lymphomas (TZL)).

Percentage, nFSC and CD21-nMFI values of neoplastic cells in B-cell lymphomas are reported in Table 2.

**Figure 5:**
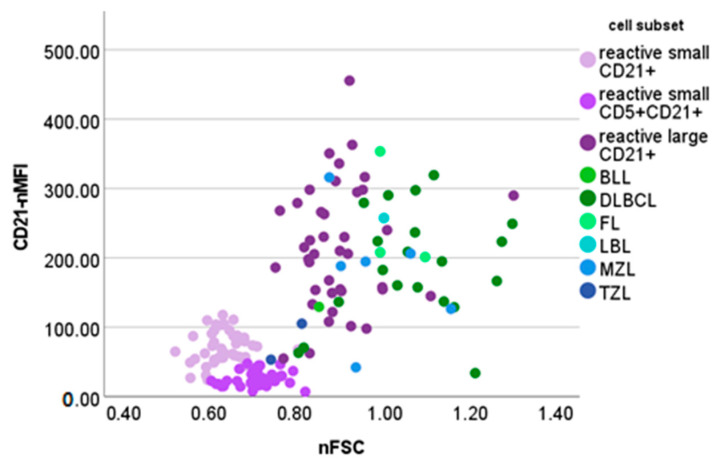
Normalized forward scatter (nFSC) and median fluorescence intensity of CD21+ cells (CD21-nMFI) values of different cellular populations detected via flow cytometry in lymph node aspirates from 42 dogs with nodal reactive hyperplasia, 31 dogs with B-cell lymphoma and 2 dogs with T-zone lymphoma. BLL = Burkitt-like lymphoma; DLBCL = diffuse large B-cell lymphoma; FL = follicular lymphoma; LBL = lymphoblastic lymphoma; MZL = marginal-zone lymphoma; TZL = T-zone lymphoma. Normalization was obtained by dividing the FSC or MFI of lymphocyte subset by the FSC or MFI of neutrophils in paired blood samples.

Figure 5 shows the nFSC and CD21-nMFI of reactive and neoplastic CD21+ cells.

**Figure 6:**
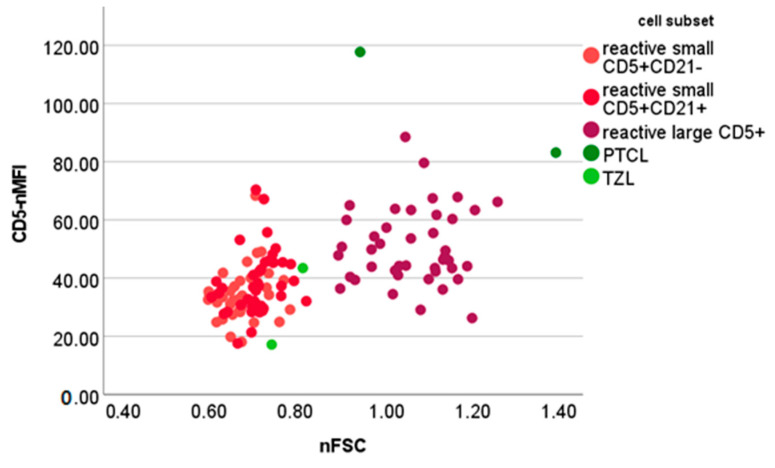
Normalized forward scatter (nFSC) and median fluorescence intensity of CD5+ cells (CD5-nMFI) values of different cellular populations detected via flow cytometry in lymph node aspirates from 42 dogs with nodal reactive hyperplasia, 4 dogs with T-cell lymphoma. PTCL = peripheral T-cell lymphoma; TZL = T-zone lymphoma. Normalization was obtained by dividing the FSC or MFI of lymphocyte subset by the FSC or MFI of neutrophils in paired blood samples.

Among the PTCL samples, neoplastic cells accounted for 60.7%, 71.7% and 98.7%, respectively; their mean nFSC was 1.06 ± 0.29 (median, 0.94; min-max, 0.85–1.39). One PTCL stained negative for CD5. The CD5-nMFI of the remaining two PTCL samples was 85.15 and 117.75, respectively. Neoplastic cells in one TZL sample accounted for 88.5%, and had an nFSC of 0.73, a CD5-nMFI of 17.15 and a CD21-nMFI of 53.22. Neoplastic cells in the second TZL sample accounted for 63.5%, and had an nFSC of 0.81, a CD5-nMFI of 43.45 and a CD21-nMFI of 105.17. Figure 6 shows the nFSC and CD5-nMFI of reactive and neoplastic CD5+ cells.

## 4. Discussion

Herein, we report an FC analysis of reactive LNs in dogs. In all samples, five cellular populations were retrieved, differing in cell size and level of antigen expression: small CD21+, large CD21+, small CD5+CD21-, small CD5+CD21+, and large CD5+ cells. Small CD5+ cells had a higher nFSC than small CD21+ cells, although only CD5+CD21+ cells reached a statistically significant difference compared to small CD21+ cells. In all cases but one, small CD21+ cells had an nFSC value < 0.74, which is the lowest value encountered in large CD21+ cells. Thus, a 0.74 nFSC was considered as a cut-off to discriminate between small and large CD21+ cells. Conversely, no overlap was found in the nFSC values of small and large CD5+ cells, and a 0.85 nFSC was considered as a cut-off to discriminate between small and large CD5+ cells. These results support the use of lineage-specific cut-offs when reporting cell size via FC. It is important to pinpoint that the flow cytometric determination of cell size (FSC) depends on the scattering properties of light when cells pass through the laser beam, and on the specific characteristics of the optical bench [18]. Thus, the size definition of cells by FSC can differ from pure morphologic (cytologic or histologic) evaluation.

Large cells showed higher MFI values compared to lineage-related small cells. This result is not unexpected, as it has been demonstrated that fluorescence intensity is also influenced by cell size [19].

Unlike what typically happens with large-cell lymphomas, discriminating between small and large lymphocytes was often difficult in the reactive lymph nodes when looking at the morphological plot (FSC vs. SSC). The identification of the two populations was easier when looking at the size combined with the CD5 or CD21 expression (Figure S3).

Small-sized CD21+ cells exceeded 60% in 3 out of 42 samples (7.1%). PARR results were not available for these cases and small cell lymphoma was excluded because it met inclusion criteria n.1 and 4. By convention, lymphoma is diagnosed if ≥60% of the cells share the same immunophenotype [2]: accordingly, some reactive LNs may be misdiagnosed as small B-cell lymphoma, potentially leading to unnecessary chemotherapeutic treatment. Unfortunately, no small B-cell lymphoma was included in the present study, thus preventing the comparison of nFSC and nMFI values between reactive and neoplastic cells. The future validation of a larger number of antibodies directed against canine B-cell antigens may prove useful to better characterize this population in both reactive and neoplastic conditions. Meanwhile, histopathology should be suggested when facing an FC pattern with a prevalence of small B-cells.

Large CD21+ cells accounted for up to 20% of the whole nodal population in our case series. Similar percentages were obtained in one DLBCL case and in the only incidence of B-lymphoblastic lymphoma. In both cases, cytologic examination revealed a prevalent population of neoplastic cells, and only a few mature residual lymphocytes. This finding may be attributable to the poor quality of the aspirate intended for FC, which is not completely representative of the LN population. FC analyses should never substitute cytologic evaluation, which should, in turn, be considered as a first mandatory step in the approach to dogs with lymphadenomegaly. To avoid misinterpretation, we strongly recommend to always interpret the nodal FC pattern in the context of the relative cytologic presentation. When examining B-cell lymphomas in our case series, neoplastic cells always had a >0.74 nFSC value and were therefore classified as “large”. Interestingly, in most of the samples, neoplastic cells had an even higher nFSC value than those of large B-cells in reactive LNs, despite retaining a similar CD21-nMFI (Figure 5). The presence of >20% large CD21+ cells and an increase in their mean nFSC are concerning regarding lymphoma diagnoses and warrant further investigation.

Different results were obtained when considering large CD5+ cells that were poorly represented in reactive LNs, accounting for a maximum of 4% of the total nucleated cells. The neoplastic cells obtained from all PTCLs (but not from TZL) were classified as “large”. In all of them, neoplastic cells accounted for more than 60% and had similar nFSC values to those of large CD5+ cells in reactive LNs. One PTCL stained negative for CD5, whereas the remaining two had a higher CD5-nMFI than their reactive counterpart. Thus, differently from large B-cell lymphoma, the presence of >4% large CD5+ cells and an increase in their median CD5-nMFI are concerning for lymphoma and warrant further investigation. Still, due to the low number of lymphoma cases, our results should only be considered as preliminary, and a larger caseload is needed for confirmation.

This is the first report of a CD5+CD21+ cell subset in non-neoplastic conditions in dogs. They appear to be CD45+ T-cells because of the concomitant expression of CD3 (Figure S2) and warrant further consideration. First, the co-expression of these two markers should not always be considered aberrant and thus diagnostic for neoplasia, as was the case in the past [20,21]. Second, the nMFI of both CD5 and CD21 was different from those of the remaining small cells, further supporting the distinct nature of CD5+CD21+ cells. Specific immunologic studies should be performed to assess their role in non-neoplastic conditions. Finally, the co-expression of CD5 and CD21 was found in both TZL cases in the present study, in line with the published literature [20,22]. When compared with the small CD5+CD21+ cells in reactive LNs, however, neoplastic cells in TZL lacked CD45 expression (data not shown) and tended to have a higher nFSC and higher nMFI of CD21. An overall estimation of the whole phenotypic pattern, as well as of cell size and antigen-specific MFI, is needed to discriminate between non-neoplastic and neoplastic (TZL) CD5+CD21+ small cells.

Concerning Ki67%, one recent study reported a mean value of 5% in normal canine LNs (maximum value, 8.1%) [8]. In our caseload, we encountered a slightly higher proliferative activity, reaching about 15%. Interestingly, a similar Ki67% was obtained within the residual small cell population in lymphomatous LNs [8] and within the B-cell population of normal LNs [15]. The differences in Ki67% between our results and those published on normal LNs are likely attributable to the different inclusion criteria and gating strategy used. Indeed, we included only enlarged, reactive LNs, thus excluding normal/physiological conditions. Furthermore, we calculated the Ki67% of the total nucleated cells, rather than limiting the calculation to the B-cell population; thus, our results cannot be used to confirm those reported by Rout et al. [15].

Irrespective of these differences, in all studies, the Ki67% values encountered in non-neoplastic lymphoid cells largely overlap with those obtained in low-grade lymphomas [13,15,23]. Of note, few non-neoplastic samples had a Ki67% exceeding the 12.2% cut-off normally used to discriminate between low- and high-grade lymphomas [10]. This finding supports the need to confirm the diagnosis of lymphoma prior to the determination of the proliferative activity, rather than considering a Ki67% > 12.2% as diagnostic for high-grade lymphoma.

The retrospective nature of the present study represents its major limitation, since it affected both the list of diagnostic tests performed in each case and the antibody panel applied to each sample. Indeed, the cause of nodal reactivity was definitively identified only in a minority of cases, preventing us from comparing the lymphoid cells’ composition of different diseases. In addition, only a few cases of lymphoma were confirmed and classified via histopathology, thus limiting the number of cases that were enrolled. Finally, we limited MFI analyses to CD5 and CD21, since further antigens were tested in only a subset of cases, with unstandardized antibody cocktails and fluorescence panels.

## 5. Conclusions

In conclusion, we describe the FC cell size and fluorescence intensity of lymphoid subpopulations in canine reactive and neoplastic LNs. Based on our results, B- and T-lymphocytes show different sizes, and specific cut-offs should be used to classify small and large cells based on the lineage of origin. In addition, we propose different criteria to diagnose large cell lymphoma, considering the percentage of large B-cells and their nFSC to diagnose large B-cell lymphoma, and the percentage of large T-cells and their CD5-nMFI to diagnose large T-cell lymphoma, respectively. As stated above, different results can be obtained when determining cell size via FC or cytology. This is why some lymphoma subtypes commonly classified as medium-sized cells via cytology (i.e., MZL) were finally classified as “large cells” via FC. This consideration further strengthens the mutual complementarity of FC and cytology in the diagnosis and classification of lymphomas.

Proliferative activity in reactive LNs is slightly higher than in normal LNs; it largely overlaps with low-grade lymphomas and occasionally with high-grade lymphomas. Finally, we describe a CD5+CD21+ small-sized cellular population in reactive LNs and suggest possible criteria to discriminate them from TZL cells. Future studies should address the biological peculiarities of this cell subset.

## Figures and Tables

**Table 1:** Percentage, normalized forward-scatter (nFSC) and median fluorescence intensity (nMFI) values in different cellular populations detected via flow cytometry in nodal aspirates from 42 dogs with nodal reactive hyperplasia. Normalization was obtained by dividing FSC or MFI of lymphocyte subset by FSC or MFI of neutrophils in paired blood samples.

Cellular Population	Mean ± Standard Deviation	Median	Min	Max
	Percentage			
Small CD21+	31.7 ± 15.1	29.2	3.9	68.9
Small CD5+	53.1 ± 15.8	54.1	5.4	85.0
Small CD5+CD21-	42.8 ± 15.1	44.2	4.7	79.4
Small CD5+CD21+	10.2 ± 9.3	8.7	0.3	55.0
Large CD21+	5.7 ± 4.0	5.0	0.5	19.3
Large CD5+	1.6 ± 1.0	1.3	0.3	4.0
	**nFSC**			
Small CD21+	0.62 ± 0.05	0.62	0.51	0.80
Small CD5+CD21-	0.67 ± 0.05	0.67	0.59	0.78
Small CD5+CD21+	0.70 ± 0.05	0.70	0.59	0.81
Large CD21+	0.90 ± 0.10	0.88	0.74	1.30
Large CD5+	1.06 ± 0.10	1.07	0.89	1.26
	**CD21-nMFI**			
Small CD21+	70.48 ± 23.07	66.39	24.07	117.70
Large CD21+	216.07 ± 88.42	207.94	54.85	455.48
Small CD5+CD21+	25.55 ± 10.35	22.70	7.01	47.51
	**CD5-nMFI**			
Small CD5+CD21-	34.54 ± 8.78	33.10	18.05	68.34
Small CD5+CD21+	38.53 ± 10.63	37.05	17.57	70.40
Large CD5+	50.68 ± 13.04	47.60	26.27	88.53

**Table 2:** Percentage, normalized forward-scatter (nFSC) and median fluorescence intensity (nMFI) values of the neoplastic population detected via flow cytometry in different B-cell lymphomas. Normalization was obtained dividing FSC or MFI of lymphocyte subset by FSC or MFI of neutrophils in paired blood samples. Numbers in brackets refer to individual cases. DLBCL = diffuse large B-cell lymphoma; MZL = marginal-zone lymphoma; FL = follicular lymphoma; LBL = lymphoblastic lymphoma; BLL = Burkitt-like lymphoma.

B-Cell Lymphoma	Mean ± Standard Deviation	Median	Min	Max
	Percentage			
DLBCL (n = 20)	76.9 ± 19.9	85.6	24.0	95.5
MZL (n = 6)	85.1 ± 9.3	87.1	67.0	93.0
FL (n = 3)	--	(60.0)	(59.0)	(89.4)
LBL (n = 1)	--	(22)	--	--
BLL (n = 1)	--	(91.4)	--	--
	**nFSC**			
DLBCL (n = 20)	1.07 ± 0.14	1.07	0.80	1.30
MZL (n = 6)	0.98 ± 0.11	1.94	0.87	1.15
FL (n = 3)	1.02 ± 0.06	0.99	0.99	1.09
LBL (n = 1)	--	(1.00)	--	--
BLL (n = 1)	--	(0.85)	--	--
	**CD21-nMFI**			
DLBCL (n = 20)	187.98 ± 79.89	188.67	33.82	319.25
MZL (n = 6)	178.93 ± 90.96	191.42	42.06	316.6
FL (n = 3)	254.22 ± 86.06	207.90	201.24	353.52
LBL (n = 1)	--	(257.40)	--	--
BLL (n = 1)	--	(129.31)	--	--

## Data Availability

Raw data of the present study are available from the corresponding author upon reasonable request.

## Supplementary Materials

The following supporting information can be downloaded at: https://www.mdpi.com/article/10.3390/vetsci10060374/s1, Figure S1: Reactive Lymph node. Ki67 analysis; Figure S2: Reactive lymph node; Figure S3: Discrimination between small and large cell populations in reactive lymph nodes and lymphomas.
