# Supplementary material for: Flow Cytometric Features of B- and T-Lmphocytes in Reactive Lymph Nodes Compared to Their Neoplastic Counterparts in Dogs
Source: Vet Sci. 2023 May 26;10(6):374. doi: 10.3390/vetsci10060374 (PMC10305363; doi:10.3390/vetsci10060374)
Supplement: Supplementary file 1 [file vetsci-10-00374-s001.zip › Figure S1.pdf]

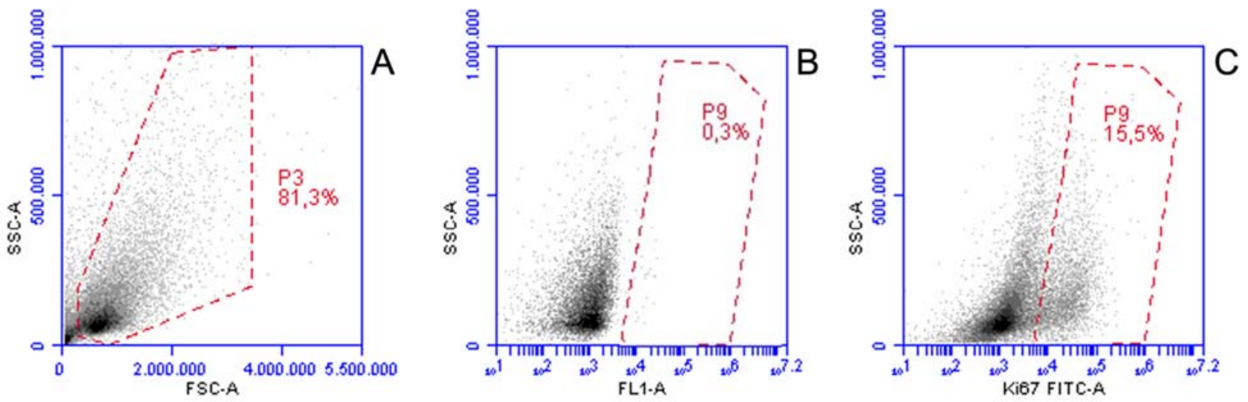

Figure S1. Reactive Lymph node. Ki67 analysis. (**A**) FSC vs SSC plot showing whole population after doublets exclusion. (**B**) isotype-stained tube; P3 events are displayed. (**C**) Ki67-stained tube; P3 events are displayed.
