# Supplementary material for: Flow Cytometric Features of B- and T-Lmphocytes in Reactive Lymph Nodes Compared to Their Neoplastic Counterparts in Dogs
Source: Vet Sci. 2023 May 26;10(6):374. doi: 10.3390/vetsci10060374 (PMC10305363; doi:10.3390/vetsci10060374)
Supplement: Supplementary file 1 [file vetsci-10-00374-s001.zip › Figure S2.pdf]

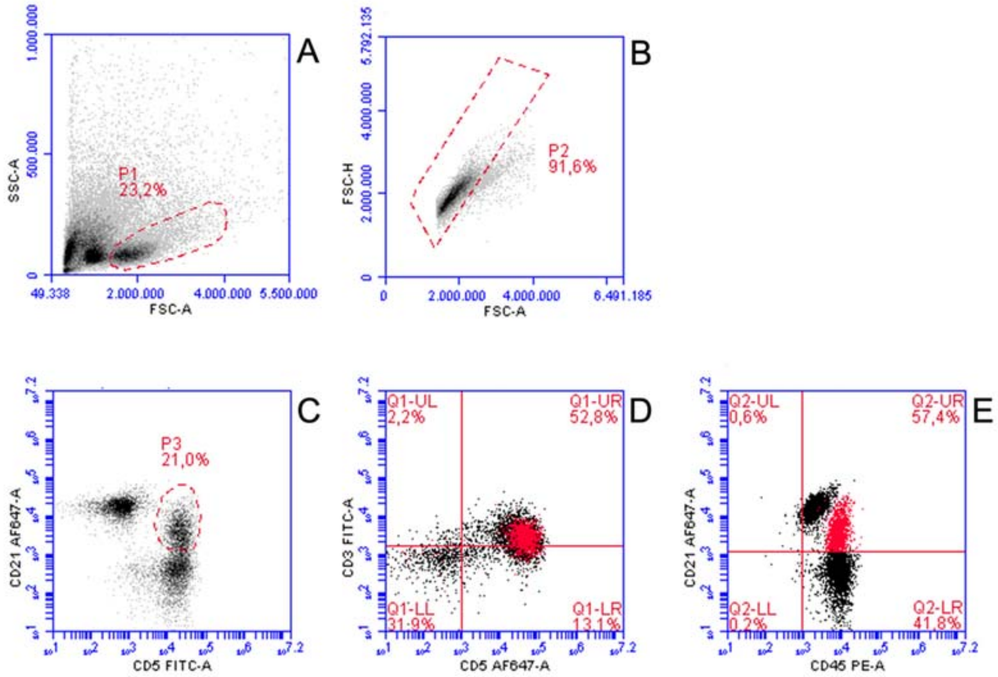

Figure S2. Reactive lymph node. (**A**) All events are displayed; a gate (P1) was set on FSC versus SSC scattergram to exclude platelets and debris. (**B**) P1 events are displayed; a second gate (P2) was set on FSC-H versus FSC-A scattergram to exclude doublets. (**C**) P2 events are displayed; a gate was set to highlight CD5+CD21+ events (P3). (**D,E**) P3 events are displayed; CD5+CD21+ cells (red dots) are CD3+ (D) and CD45+ (E).
