# Supplementary material for: Flow Cytometric Features of B- and T-Lmphocytes in Reactive Lymph Nodes Compared to Their Neoplastic Counterparts in Dogs
Source: Vet Sci. 2023 May 26;10(6):374. doi: 10.3390/vetsci10060374 (PMC10305363; doi:10.3390/vetsci10060374)
Supplement: Supplementary file 1 [file vetsci-10-00374-s001.zip › Figure S3.pdf]

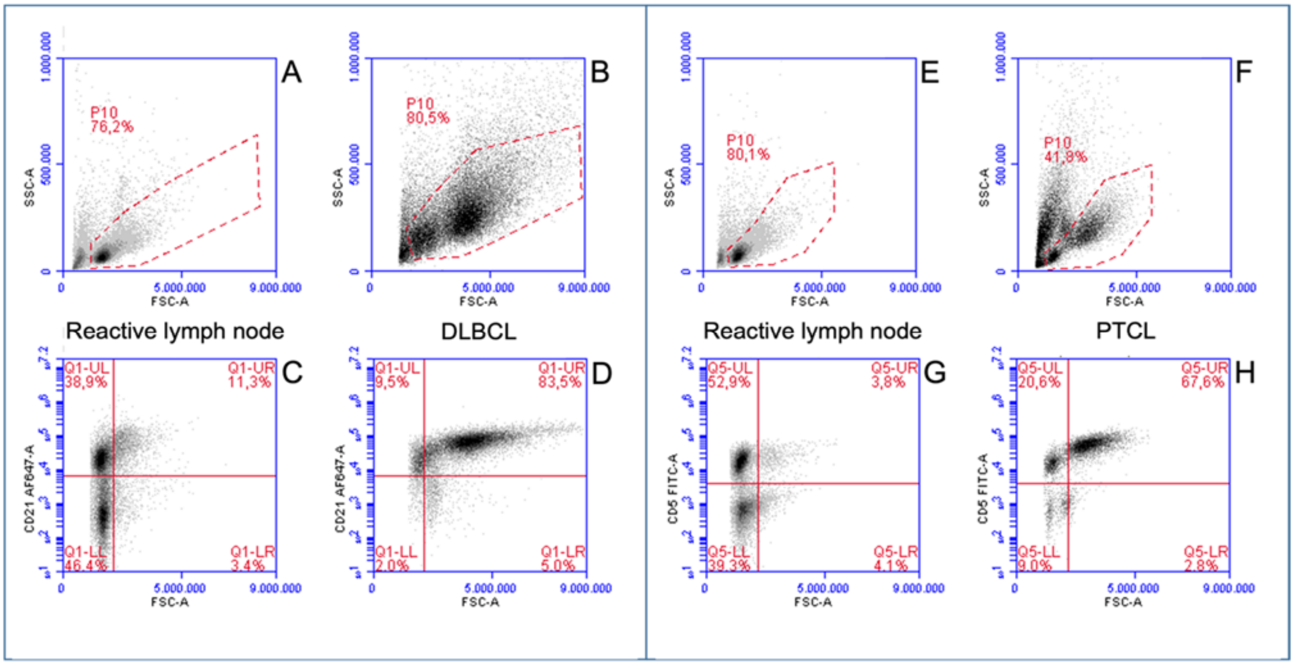

Figure S3. Discrimination between small and large cell populations in reactive lymph nodes (**A, C, E, G**) and lymphomas (**B, D, F, H**). (**A, B, E, F**) FSC vs SSC plot showing whole population after doublets exclusion. (**C, D, G, H**) P10 events of the above plots are displayed. While in reactive lymph nodes there is often continuum between the small and large cells (**A, E**) in lymphomas they are more clearly separated (**B, F**). The discrimination between the two populations is made easier by using CD21 (**C, D**) or CD5 expression (**G, H**). DLBCL=Diifuse Large B-Cell Lymphoma; PTCL: Peripheral T-Cell Lymphoma.
